# Improvements in no evidence of disease activity with ublituximab vs. teriflunomide in the ULTIMATE phase 3 studies in relapsing multiple sclerosis

**DOI:** 10.3389/fneur.2024.1473284

**Published:** 2024-10-24

**Authors:** Enrique Alvarez, Lawrence Steinman, Edward J. Fox, Hans-Peter Hartung, Peiqing Qian, Sibyl Wray, Derrick Robertson, Krzysztof Selmaj, Daniel Wynn, Koby Mok, Yihuan Xu, Karthik Bodhinathan, Hari P. Miskin, Bruce A. C. Cree

**Affiliations:** ^1^Department of Neurology, University of Colorado, Aurora, CO, United States; ^2^Beckman Center for Molecular Medicine, Stanford University, Stanford, CA, United States; ^3^TG Therapeutics, New York, NY, United States; ^4^Department of Neurology, Medical Faculty, Heinrich Heine University Düsseldorf, Düsseldorf, Germany; ^5^Brain and Mind Centre, University of Sydney, Sydney, NSW, Australia; ^6^Medical University of Vienna, Vienna, Austria; ^7^Palacký University Olomouc, Olomouc, Czechia; ^8^Swedish Neuroscience Institute, Seattle, WA, United States; ^9^Hope Neurology, Knoxville, TN, United States; ^10^Department of Neurology, University of South Florida, Tampa, FL, United States; ^11^Center of Neurology, Łódź, Poland; ^12^Department of Neurology, University of Warmia and Mazury, Olsztyn, Poland; ^13^Consultants in Neurology, Northbrook, IL, United States; ^14^Department of Neurology, UCSF Weill Institute for Neurosciences, University of California, San Francisco, San Francisco, CA, United States

**Keywords:** anti-CD20, disability, disease activity, disease-modifying therapy, multiple sclerosis, no evidence of disease activity, relapse, BRIUMVI

## Abstract

**Background:**

Ublituximab is a novel anti-CD20 monoclonal antibody glycoengineered for enhanced antibody-dependent cellular cytotoxicity. The phase 3 ULTIMATE I and II studies showed significant improvements in annualized relapse rate, total number of gadolinium-enhancing (Gd+) T1 lesions, and total number of new or enlarging T2 at Week 96, as well as improvement in the proportion of participants with no evidence of disease activity (NEDA) from Weeks 24–96 with ublituximab vs. teriflunomide.

**Methods:**

In ULTIMATE I (NCT03277261; www.clinicaltrials.gov) (*N* = 549) and II (NCT03277248; www.clinicaltrials.gov) (*N* = 545), participants with relapsing multiple sclerosis received ublituximab 450 mg intravenous infusion every 24 weeks (following Day 1 infusion of 150 mg and Day 15 infusion of 450 mg) or teriflunomide 14 mg oral once daily for 96 weeks. Pooled *post hoc* analyses evaluated NEDA by treatment epoch and participant subtype: age ( ≤ 38 or >38 years), early or later disease (<3 or ≥3 years following diagnosis), treatment history (treatment naïve or previously treated), 0 or ≥1 Gd+ T1 lesions at baseline, and Expanded Disability Status Scale score ≤ 3.5 or >3.5 at baseline. NEDA was defined as no confirmed relapses, no Gd+ T1 lesions, no new or enlarging T2 lesions, and no disability progression confirmed for ≥12 weeks.

**Results:**

NEDA rates in the ublituximab vs. teriflunomide cohorts by treatment epoch were: Weeks 0–96, 44.6% vs. 12.4% (3.6 × improvement); Weeks 24–96 (re-baselined), 82.1% vs. 22.5% (3.6 × improvement); and Weeks 48–96 (re-baselined), 88.2% vs. 30.4% (2.9 × improvement) (all *p* < 0.0001). The primary driver of disease activity in ublituximab-treated participants was new or enlarging T2 lesions during Weeks 0–24. 41.8% of ublituximab-treated participants who had evidence of disease activity in the first year (Weeks 0–48) experienced NEDA in the second year of treatment (Weeks 48–96) compared with 17.3% of teriflunomide-treated participants. At Weeks 24–96 (re-baselined), rates of NEDA were significantly higher with ublituximab than teriflunomide in all participant subtypes (all *p* < 0.0001).

**Conclusions:**

ULTIMATE I and II pooled *post hoc* analyses demonstrated a consistent NEDA benefit among ublituximab-treated participants across treatment epochs and key participant subpopulations.

## 1 Introduction

With recent approvals of new highly effective therapies and the shifting paradigm of using such agents earlier in the course of relapsing multiple sclerosis (RMS) treatment, suppression of measurable disease activity is becoming an important goal both in clinical trials and in clinical practice (1–4). Accumulating evidence suggests that initial treatment with a more efficacious disease-modifying therapy (DMT), including anti-CD20 agents that mediate B-cell depletion, may reduce the risk of relapse and disability worsening and improve long-term outcomes (5–11).

A commonly used metric of disease control is the 3-parameter no evidence of disease activity (NEDA-3), defined as an absence of disease activity due to relapses and magnetic resonance imaging (MRI) lesions (gadolinium-enhancing [Gd+] lesions per T1-weighted MRI scan [Gd+ T1 lesions] and new or enlarging hyperintense lesions per T2-weighted MRI scan [T2 lesions]) as well as no sustained disease progression as measured by the Expanded Disability Status Scale (EDSS) (3, 12–15). This measure captures both focal MRI inflammatory activity and functional disability worsening. NEDA-3 was incorporated as an outcome in clinical trials and proposed as a therapeutic goal in clinical practice, although some clinicians and researchers questioned the practicality of NEDA as a therapeutic goal using currently available DMTs (2, 12, 14, 16, 17). In support of NEDA as a potential treatment goal, a meta-analysis of 27 clinical studies, including 11 studies of high efficacy therapies, reported that NEDA-3 was significantly associated with no long-term disability progression in RMS (18), highlighting the importance of head-to-head comparison of NEDA rates between high- vs. moderate-efficacy therapies like ublituximab and teriflunomide in a controlled trial.

**Figure 1:**
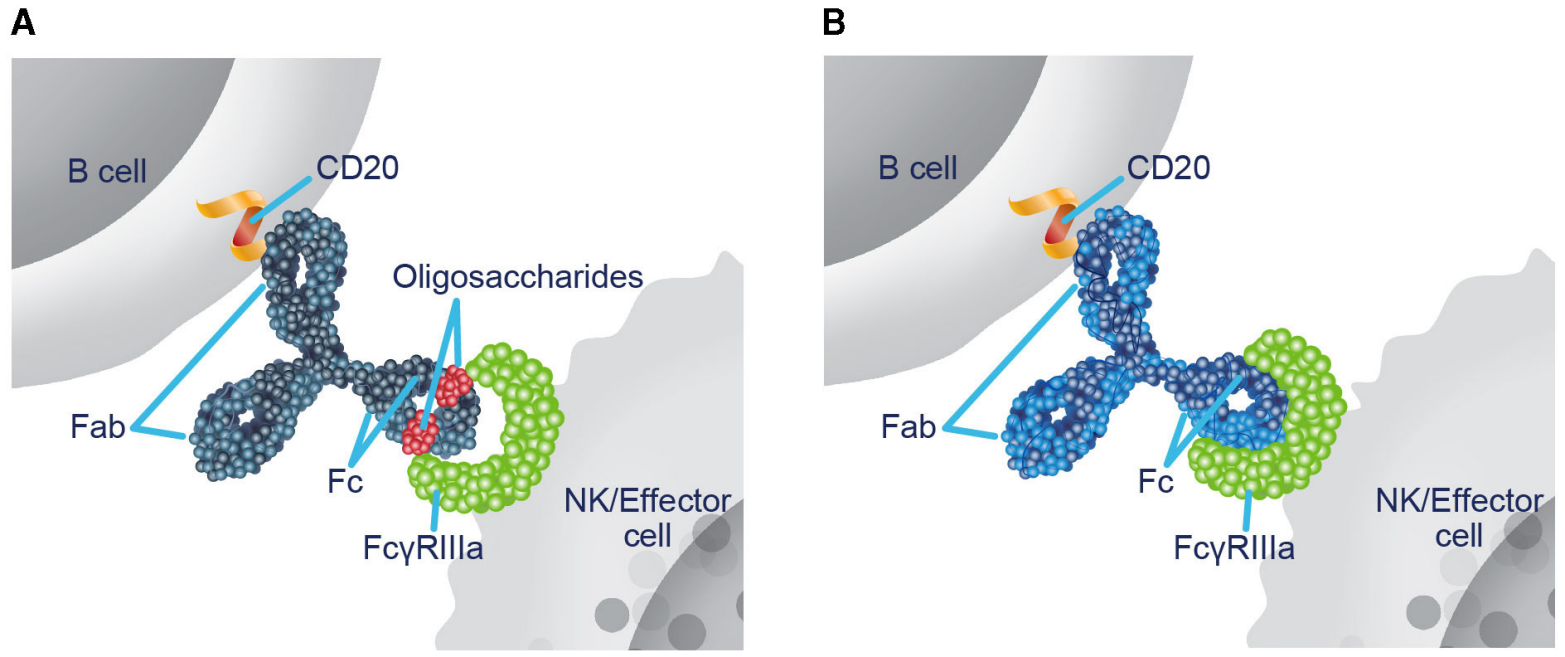
Ublituximab is glycoengineered to enhance ADCC. **(A)** In nonglycoengineered anti-CD20 antibodies, the core fucose of Fc-linked oligosaccharides sterically blocks interaction with FcγRIIIa, reducing affinity (22, 23). **(B)** Ublituximab is glycoengineered to have a low fucose content in the Fc region, which allows for closer interaction and enhanced affinity for all variants of FcγRIIIa (20, 23, 24). ADCC, antibody-dependent cellular cytotoxicity; Fab, fragment antigen-binding; Fc, fragment crystallizable; FcγRIIIa, Fc gamma receptor IIIa; NK, natural killer.

Ublituximab is a novel monoclonal antibody (mAb) that targets a unique epitope of CD20 on B cells and is glycoengineered to enhance antibody-dependent cellular cytotoxicity (ADCC) (19–21). Nonglycoengineered anti-CD20 antibodies have a reduced affinity for fragment crystallizable (Fc) gamma receptor IIIa (FcγRIIIa), as the core fucose of Fc-linked oligosaccharides sterically hinders interaction with FcγRIIIa (22, 23). The low fucose content in the Fc region of ublituximab enables closer interaction and greater affinity for all variants of FcγRIIIa (Figure 1) (19–21, 23, 24). In preclinical studies, ublituximab demonstrated 25- to 30-fold greater ADCC potential relative to ocrelizumab and ofatumumab and > 2,000-fold greater than that of rituximab (25, 26). Compared with other infused anti-CD20 therapies, ublituximab is administered in lower doses and with shorter infusion times after the first infusion (24, 27–30).

The phase 3 ULTIMATE I and ULTIMATE II studies evaluated the efficacy and safety of ublituximab, an anti-CD20 mAb known to deplete B cells, vs. teriflunomide, a dihydroorotate dehydrogenase–inhibitor known to limit proliferation of activated lymphocytes, in participants with RMS (31). These studies met their primary endpoint, demonstrating a statistically significant reduction in annualized relapse rate with ublituximab compared with teriflunomide (59% [*p* ≤ 0.001] and 49% [*p* = 0.002] relative reduction) as well as significant improvements in the mean number of Gd+ T1 lesions (97% and 96% relative reduction; *p* < 0.001 for both studies) and number of new or enlarging T2 lesions (92% and 90%; *p* < 0.001 for both studies). In a prespecified analysis of both studies, a higher proportion of participants treated with ublituximab than with teriflunomide experienced NEDA from Weeks 24 to 96 (inclusive of MRI disease activity at Week 24; 44.6% vs. 15.0% in ULTIMATE I and 43.0% vs. 11.4% in ULTIMATE II).

The current analyses were performed to further characterize the effects of ublituximab on NEDA-3 using data pooled across the phase 3 ULTIMATE studies.

## 2 Materials and methods

### 2.1 Study design and participants

*Post hoc* analyses characterized NEDA-3 in the pooled population of two identical, phase 3, randomized, multicenter, double-blind, double-dummy, active-controlled studies, ULTIMATE I (ClinicalTrials.gov identifier: NCT03277261) and ULTIMATE II (NCT03277248), which were conducted at 104 sites across 10 countries. The study protocols were approved by the institutional review board or ethics committee at each study site and conformed to Good Clinical Practice guidelines and the principles of the Declaration of Helsinki. All participants provided written informed consent. Details of the study methods were previously reported (31). Briefly, the studies enrolled participants aged 18–55 years with RMS (relapsing-remitting or secondary-progressive multiple sclerosis [MS]) who had ≥ 2 relapses in the previous 2 years or 1 relapse and/or ≥ 1 Gd+ T1 lesions in the year prior to screening, brain abnormalities on MRI consistent with MS, an EDSS score of 0.0–5.5 at screening, and neurologic stability for ≥ 30 days before screening and baseline. Key exclusion criteria were a diagnosis of primary-progressive MS, previous anti-CD20 or other B-cell–directed treatment, and disease duration ≥ 10 years from onset with an EDSS score ≤ 2.0 at screening. Participants were randomized 1:1 to receive intravenous infusions of ublituximab (150 mg infused over 4 h on Day 1; 450 mg infused over 1 h on Day 15 and at Weeks 24, 48, and 72) with oral placebo or oral teriflunomide 14 mg once daily for 96 weeks with intravenous placebo.

### 2.2 Clinical and MRI endpoints

Clinical evaluations, including EDSS, were performed at baseline and every 12 weeks. Protocol-defined relapses included new or worsening neurological symptoms that were attributable to MS only in the absence of fever or infection, persisted for > 24 h, were immediately preceded by a stable or improving neurological state for ≥30 days, and were accompanied by objective neurological worsening consistent with at least a half-point increase on the EDSS, 2.0-point increase in 1 EDSS functional system score, or 1.0-point increase in each of ≥2 EDSS functional system scores. All relapses were centrally confirmed via an independent relapse adjudication panel. Confirmed disability progression (CDP) was defined as a ≥1.0-point increase from baseline in EDSS score not attributable to another etiology (e.g., fever, concurrent illness, or concomitant medication) when the baseline score was ≤ 5.5 and an increase of ≥0.5 point when the baseline score was >5.5 that was sustained and confirmed for ≥12 weeks after the initial documentation of neurological worsening. Brain MRI assessments were performed at Weeks 12 (Gd+ T1 lesions) and Weeks 24, 48, and 96 (Gd+ T1 lesions, T2 lesions, and brain volume). NEDA-3 was defined as no confirmed relapses, no Gd+ T1 lesions, no new or enlarging T2 lesions, and no 12-week CDP.

### 2.3 Statistical analyses

*Post hoc* analyses evaluated NEDA-3 by treatment epoch and participant subtype, including age ( ≤ 38 or >38 years), early and later disease (<3 and ≥3 years following diagnosis, respectively), treatment naïve or previously treated, 0 or ≥1 Gd+ T1 lesions at baseline, and EDSS score ≤ 3.5 or >3.5 in a prespecified modified intention-to-treat population that included all participants who received ≥1 dose of trial drug and had 1 baseline and ≥1 postbaseline efficacy and MRI assessment. The NEDA rate was the proportion of participants with NEDA-3, excluding participants who discontinued treatment early due to reasons other than death and lack of efficacy (Supplementary Table 1) during the analysis time frame, similar to prior methodology used for NEDA analysis (32). For re-baselined epochs, all components of NEDA-3 were re-baselined to Week 24 or Week 48 as indicated. EDSS progression events that occurred at the last scheduled visit (Week 96) during the timeframe were not included as 12-week CDP due to their inability to be confirmed 12 weeks later. *P* values and odds ratio (OR) were derived from a logistic regression model with adjustments for treatment, study, region, baseline EDSS strata, and log-transformed baseline MRI lesion counts (T1 nonenhancing, T2, and Gd+ T1 lesions). Assessment of Gd+ T1 and T2 lesions and relapses in the past 1 or 2 years between participants with and without NEDA-3 was based on *t* test for continuous variables and chi-square test or Fisher's exact test for categorical variables. Assessments of Gd+ T1 and T2 lesions and relapses in the past 1 or 2 years between participants with and without NEDA-3 were based on *t* test for continuous variables and chi-squared test or Fisher's exact test for categorical variables.

## 3 Results

### 3.1 Participant demographics and disease characteristics

ULTIMATE I and ULTIMATE II enrolled a total of 1,094 participants (ublituximab, *n* = 546; teriflunomide, *n* = 548) (31). Demographic and disease characteristics of participants included in the pooled NEDA-3 analyses (teriflunomide, *n* = 524; ublituximab, *n* = 520) were well balanced across treatment arms (Table 1). Table 2 shows demographic and disease characteristics of ublituximab-treated participants who did or did not experience NEDA-3 during the 2-year study period (Weeks 0–96). When compared with participants who did not experience NEDA-3, participants who experienced NEDA-3 were more likely to be free of Gd+ T1 lesions (70.3% vs. 36.8%; *p* < 0.0001) and other indications of less active disease at baseline, e.g., on average, fewer relapses in the past 1 (mean 1.2 ± 0.5 vs. 1.4 ± 0.7; *p* = 0.0018) or 2 years (mean 1.7 ± 0.8 vs. 1.9 ± 1.1; *p* = 0.0022), fewer T2 lesions (mean 55.3 ± 35.5 vs. 72.4 ± 41.5; *p* < 0.0001), and a smaller T2 lesion volume (mean 12.8 ± 14.3 mL vs. 17.2 ± 14.9 mL; *p* = 0.0007) than those who did not experience NEDA-3.

**Table 1:** Participant demographics and baseline characteristics.^a^

**Characteristic**	**Evaluated population**	
**Mean** ±**standard deviation or %**	**Teriflunomide (*****n*** = **524)**	**Ublituximab (*****n*** = **520)**
Age, years	36.5 ± 9.4	35.3 ± 8.7
Sex, female, %	64.3	63.3
**Region, %**		
US & Western Europe	9.0	8.7
Eastern Europe	91.0	91.3
Duration of MS since first symptoms, years	7.0 ± 6.0	7.4 ± 6.4
Time since diagnosis, years	4.7 ± 5.0	4.9 ± 5.3
Number of relapses in last 12 months	1.3 ± 0.7	1.3 ± 0.6
Number of relapses in last 24 months	1.9 ± 1.0	1.8 ± 1.0
Time since most recent relapse, months	6.3 ± 4.9	7.1 ± 8.6
EDSS score at screening	2.9 ± 1.2	2.9 ± 1.3
T2 lesion volume, mL	15.2 ± 16.7	15.2 ± 14.8
Number of T2 lesions	62.5 ± 39.6	64.8 ± 39.8
Participants free of Gd+ T1 lesions, %	53.4	51.7

^a^Participants included in NEDA-3 analyses.

EDSS, Expanded Disability Status Scale; Gd+, gadolinium-enhancing; MS, multiple sclerosis; NEDA-3, 3-parameter no evidence of disease activity.

**Table 2:** Demographics and baseline characteristics in ublituximab-treated participants with or without NEDA-3 at Weeks 0–96.^a^

**Characteristic**	**Evaluated ublituximab-treated population**	
**Mean** ±**standard deviation or %**	**With NEDA-3 (*****n*** = **232)**	**Without NEDA-3 (*****n*** = **288)**
Age, years	36.5 ± 8.9	34.4 ± 8.5
Sex, female, %	62.1	64.2
**Region, %**		
US & Western Europe	6.0	10.8
Eastern Europe	94.0	89.2
Duration of MS since first symptoms, years	7.8 ± 6.7	7.1 ± 6.1
Time since diagnosis, years	5.1 ± 5.5	4.7 ± 5.1
Number of relapses in last 12 months^*^	1.2 ± 0.5	1.4 ± 0.7
Number of relapses in last 24 months^†^	1.7 ± 0.8	1.9 ± 1.1
Time since most recent relapse, months	7.0 ± 4.9	7.3 ± 10.7
EDSS score at screening	2.8 ± 1.2	2.9 ± 1.3
T2 lesion volume, mL^‡^	12.8 ± 14.3	17.2 ± 14.9
Number of T2 lesions^§^	55.3 ± 35.5	72.4 ± 41.5
Participants free of Gd+ T1 lesions^§^, %	70.3	36.8

^*^p = 0.0018, ^†^p = 0.0022, ^‡^p = 0.0007, and ^§^p <0.0001.

^a^Participants included in NEDA-3 analyses.

EDSS, Expanded Disability Status Scale; Gd+, gadolinium-enhancing; MS, multiple sclerosis; NEDA-3, 3-parameter no evidence of disease activity.

### 3.2 NEDA outcomes

**Figure 2:**
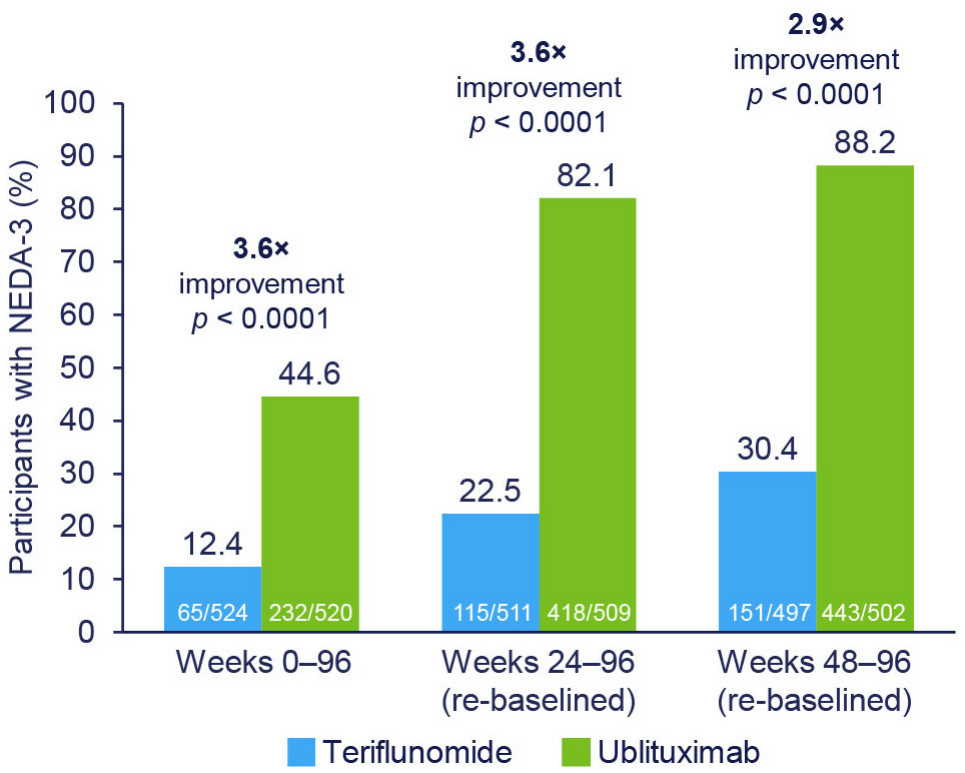
NEDA-3 rates by treatment epoch. NEDA-3 was defined as no confirmed relapses, no Gd+ T1 lesions, no new or enlarging T2 lesions, and no 12-week confirmed disability progression. Pooled *post hoc* analysis. Modified intention-to-treat population. Gd+, gadolinium-enhancing; NEDA-3, 3-parameter no evidence of disease activity.

NEDA-3 rates were significantly higher with ublituximab compared with teriflunomide during the overall treatment period (Weeks 0–96; OR [95% confidence interval (CI)] = 7.36 [5.30–10.23]; *p* < 0.0001) (Figure 2). Following re-baselining at Week 24 and Week 48, rates of NEDA-3 were 3.6-fold (Weeks 24–96; OR [95% CI] = 17.94 [12.93–24.89]; *p* < 0.0001) and 2.9-fold (Weeks 48–96; OR [95% CI] = 19.66 [13.74–28.11]; *p* < 0.0001) greater, respectively, with ublituximab than teriflunomide (Figure 2). Covariate analysis indicated that lower Gd+ T1 lesion count at baseline and geographic location tended to impact NEDA outcomes. Among ublituximab-treated participants who did not experience NEDA-3 during the first year, 210/267 (78.7%) experienced NEDA-3 during the second year, whereas only 86/422 (20.4%) of teriflunomide-treated participants who did not experience NEDA-3 during the first year did so during the second year.

**Figure 3:**
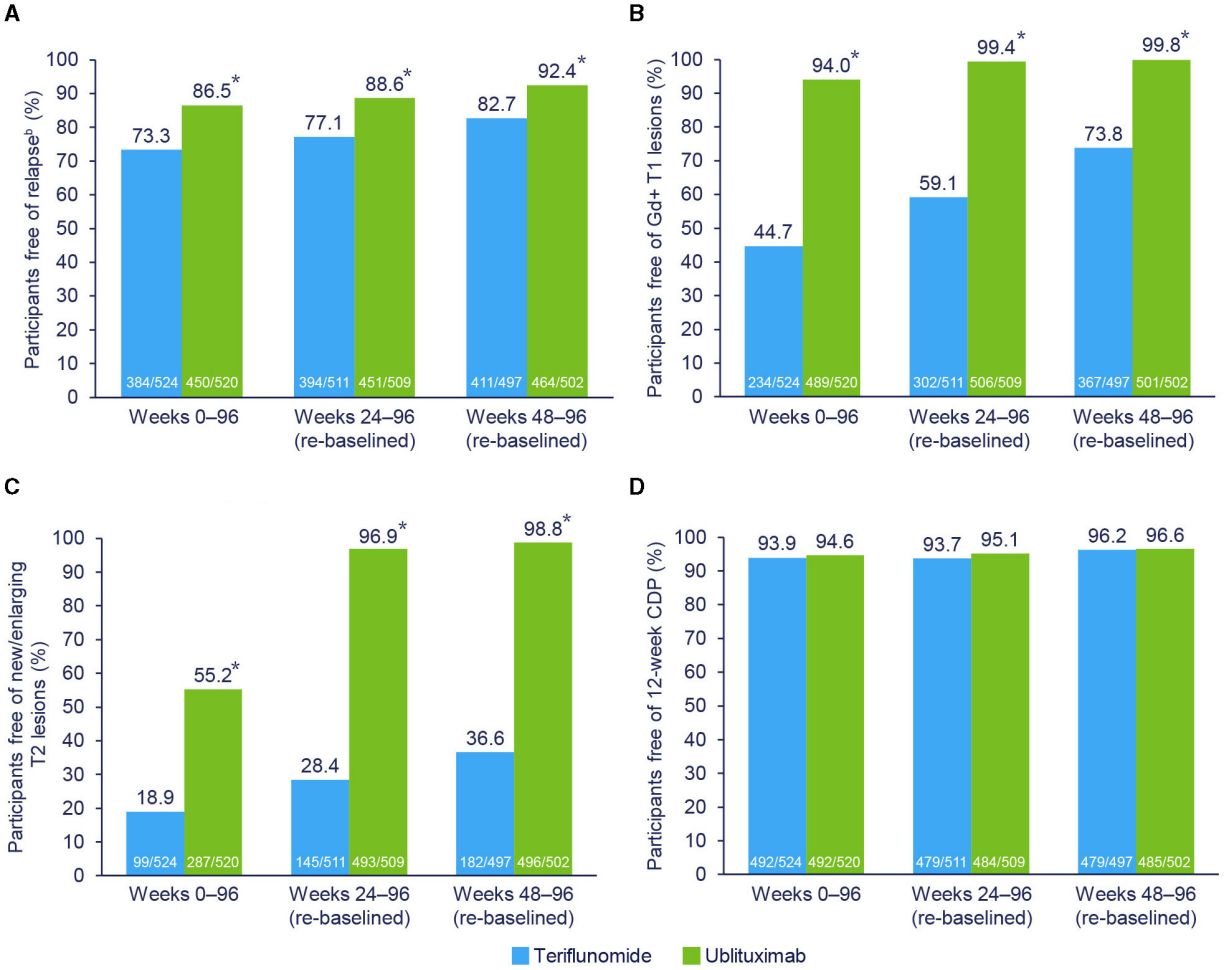
Components driving NEDA-3.^a^ *p < 0.0001. ^a^Participants may have > 1 component of evidence of disease activity. ^b^Independent Relapse Adjudication Committee confirmed. Pooled *post hoc* analysis. Modified intention-to-treat population. CDP, confirmed disease progression; Gd+, gadolinium-enhancing; NEDA-3, 3-parameter no evidence of disease activity. **(A)** Free of relapse. **(B)** Free of Gd+ T1 lesions. **(C)** Free of new/enlarging T2 lesions. **(D)** Free of 12-week CDP.

During the study (Weeks 0–96), 87.6% of teriflunomide-treated and 55.4% of ublituximab-treated participants had evidence of disease activity. Re-baselined data for Weeks 24–96 showed evidence of disease activity in 77.5% and 17.9% of teriflunomide and ublituximab treatment groups, respectively. The proportions of participants free of disease activity components during the Weeks 0–96 and Weeks 24–96 (re-baselined) epochs are shown in Figure 3. For Weeks 0–96, new or enlarging T2 lesions were the primary driver of disease activity in the teriflunomide (occurring in 81.1% of participants) and ublituximab (occurring in 44.8% of participants) groups. In contrast, the leading cause of disease activity during Weeks 24–96 (re-baselined) and Weeks 48–96 (re-baselined) was new or enlarging T2 lesions with teriflunomide (occurring in 71.6% and 63.4% of participants, respectively) and relapse with ublituximab (occurring in 11.4% and 7.6% of participants, respectively).

Sensitivity analyses that excluded MRI activity at Week 12 showed a minimal effect of Gd+ T1 lesions on NEDA-3 rates at Weeks 0–96 (ublituximab: 45.0%; teriflunomide: 12.8%). NEDA-3 rates at Weeks 0–48, excluding the Week 12 MRI, were 49.7% for ublituximab-treated participants and 21.2% for teriflunomide-treated participants (Supplementary Table 2).

**Figure 4:**
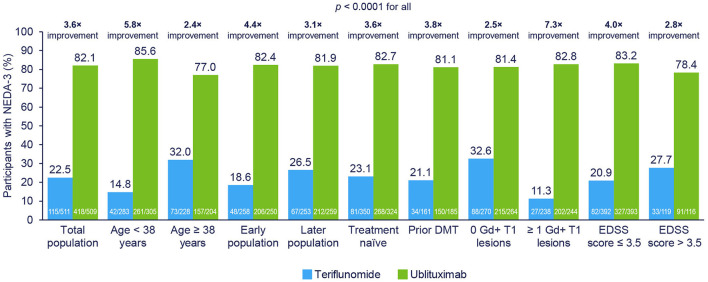
NEDA-3 at Weeks 24–96 (re-baselined) in participant subgroups. NEDA-3 was defined as no confirmed relapses, no Gd+ T1 lesions, no new or enlarging T2 lesions, and no 12-week confirmed disability progression. Early disease population vs. later disease population, defined as <or ≥ median time, was approximately 3 years from MS diagnosis to study randomization. Pooled *post hoc* analysis. Modified intention-to-treat population. DMT, disease-modifying therapy; EDSS, Expanded Disability Status Scale; Gd+, gadolinium-enhancing; NEDA-3, 3-parameter no evidence of disease activity.

As shown in Figure 4, NEDA-3 at Weeks 24–96 (re-baselined) was improved with ublituximab vs. teriflunomide among all evaluated subgroups (*p* < 0.0001 for all).

## 4 Discussion

NEDA-3 has emerged as a practical tool for characterizing therapeutic efficacy using commonly used endpoints for disease activity and worsening disability. This *post hoc* analysis showed that ublituximab consistently outperformed teriflunomide on the NEDA-3 composite outcome regardless of treatment epoch or participant subgroup, supporting the established efficacy profile of ublituximab. Rates of achieving NEDA-3 were significantly higher with ublituximab than teriflunomide at Week 96 when compared with the original baseline (Week 0) and when the baseline was redefined as Week 24 or Week 48 (all *p* < 0.0001). The re-baselined Weeks 24–96 epoch data may be more clinically meaningful than the Weeks 0–96 epoch data considering that most DMTs require several weeks or months to produce an appreciable effect on MRI parameters (2). A re-baselining approach after the anticipated onset of action of the DMT was proposed to better characterize the full efficacy of a DMT unconfounded by disease activity that is destined to occur before the DMT has had sufficient time to become fully effective (2). Previous studies implemented this approach, arguing that the corresponding results provide a more reliable indication of overall differences in efficacy between treatment arms (33, 34).

Among participants who did not experience NEDA-3 during the first year of treatment, 78.7% of those in the ublituximab group vs. only 20.4% of teriflunomide-treated participants experienced NEDA-3 during the second year. This benefit, which was observed despite higher rates of achieving NEDA-3 during Weeks 0–48 with ublituximab (49.3%) vs. teriflunomide (20.1%), supports the high rates of full efficacy associated with longer-term ublituximab treatment and the value of persisting with ublituximab for people with MS who do not attain NEDA-3 during their initial year of therapy.

New or enlarging T2 lesions and Gd+ T1 lesions were the key drivers of disease activity in teriflunomide-treated participants who did not experience NEDA during Weeks 0–96, Weeks 24–96 (re-baselined), and Weeks 48–96 (re-baselined). In contrast, among participants who received ublituximab, new or enlarging T2 lesions was the primary driver of disease activity during Weeks 0–96, but MRI activity was no longer a key driver of disease activity during Weeks 24–96 or Weeks 48–96. These observations are consistent with the strong anti-inflammatory effects as noted in MRI scans of anti-CD20 therapies (9, 35–37), which, as noted above, may require weeks to months to become fully apparent.

In the subpopulation analyses, the greatest NEDA benefit with ublituximab vs. teriflunomide was seen in who were younger, with a shorter time since MS diagnosis, with at least 1 Gd+ T1 lesion at baseline and an EDSS score ≤ 3.5 at baseline. These subpopulations are associated with higher inflammatory disease activity and these larger reductions reflect a disproportionately greater anti-inflammatory effect.

Similar analyses evaluated the effects of other anti-CD20 agents on NEDA-3. In a *post hoc* analysis of pooled data from two identical, phase 3, multicenter, randomized, double-blind, double-dummy studies (OPERA I [NCT01247324] and OPERA II [NCT01412333]), the proportion of ocrelizumab-treated participants with NEDA-3 from Week 0 to Week 96 was 47.7%, and 72.2% from Week 24 (re-baselined) to Week 96 (34). An analysis of pooled data from the phase 3 ASCLEPIOS I (NCT02792218) and ASCLEPIOS II (NCT02792231) trials found that 47.0% of ofatumumab-treated participants experienced NEDA-3 from Month 0 to Month 12, and 87.8% experienced NEDA-3 from Month 12 (re-baselined) to Month 24 (38). While the NEDA analyses from the OPERA and ASCLEPIOS trials did not include MRI assessments at Week 12, sensitivity analyses of the ULTIMATE data showed a minimal contribution of Week 12 MRI activity to NEDA-3 rates at Weeks 0–96.

Limitations of the pooled NEDA-3 data include the *post hoc* nature of the analyses based on controlled studies, which might vary from real-world clinical population. In addition, the components measured in NEDA-3 (i.e., relapses, EDSS worsening, and MRI inflammatory activity) are associated with the inflammatory phase of MS and do not characterize other important aspects of disease progression, such as cognitive and upper extremity dysfunction (3, 39, 40). Further, NEDA-3 constituents are thought to reflect the classic view of MS as a disease of white matter, whereas growing evidence suggests that MS pathology involves both demyelination and neurodegeneration (3). Expanded definitions of NEDA incorporate brain atrophy (NEDA-4) (16) and/or other components, such as fluid biomarkers (e.g., neurofilament light chain levels), cognitive function, and psychological or quality-of-life measures (2, 41–43). A brain volume loss of ≥ 0.4% per year was suggested as a cutoff value to define pathological brain atrophy in participants with MS (44). NEDA-4 may be an important measure, because brain atrophy is associated with both cognitive dysfunction (45) and long-term disability progression (44, 46–48). Finally, the safety profiles of the two study drugs could contribute to overall NEDA outcomes for patients, and future studies must account for such effects.

The concept of minimal evidence of disease activity (MEDA) may be of broader clinical use because it permits a small amount of disease activity and may be more tolerable for practitioners. As an example, an increase of 3 voxels in T2 lesions would be considered disease activity in clinical trials (37), but in clinical practice, this small change would not likely prompt treatment changes. Further, long-term observational studies failed to identify a robust contribution of minimal T2 lesion formation to long-term disability (49). However, a potential barrier to widespread adoption is that definitions of MEDA vary across studies and include (1) no relapse, ≤ 2 new T2 lesions, and no Gd+ lesions (50, 51), (2) no relapse or 1 relapse without residual disability, no MRI activity or ≤ 2 new/enlarged T2 lesions or 1 Gd+ lesion, and no EDSS disability progression confirmed at 3 months (52), and (3) ≤ 2 new T2 lesions or ≤ 1 Gd+ lesions (53). However, all were associated with reduced disability progression.

In conclusion, these data showing improved NEDA-3 rates with ublituximab compared with teriflunomide both in the overall population and in key subgroups add to the evidence of clinical benefit with ublituximab seen in the phase 3 ULTIMATE trials.

## Data Availability

The raw data supporting the conclusions of this article will be made available by the authors, without undue reservation.
