# Supplementary material for: Improvements in no evidence of disease activity with ublituximab vs. teriflunomide in the ULTIMATE phase 3 studies in relapsing multiple sclerosis
Source: Front Neurol. 2024 Oct 24;15:1473284. doi: 10.3389/fneur.2024.1473284 (PMC11542255; doi:10.3389/fneur.2024.1473284)
Supplement: Supplementary file 1 [file Table_1.docx]

Supplementary Material

**Supplementary Table 1.** Summary of participants from ULTIMATE I and II who were excluded from the NEDA analysis. Exclusion applied to mITT population for ublituximab (n=543) and teriflunomide (n=546). mITT, modified intention-to-treat; NEDA, no evidence of disease activity.

|  | Ublituximab  (n = 23) | Teriflunomide (n = 22) |
| --- | --- | --- |
| Subject withdrawal of consent | 4 | 13 |
| Adverse event | 13 | 2 |
| Investigator/sponsor decision | 2 | 4 |
| Lost to follow-up | 1 | 2 |
| Pregnancy | 2 | 0 |
| COVID-19 | 1 | 0 |
| Other | 0 | 1 |
