# Supplementary material for: Improvements in no evidence of disease activity with ublituximab vs. teriflunomide in the ULTIMATE phase 3 studies in relapsing multiple sclerosis
Source: Front Neurol. 2024 Oct 24;15:1473284. doi: 10.3389/fneur.2024.1473284 (PMC11542255; doi:10.3389/fneur.2024.1473284)
Supplement: Supplementary file 2 [file Table_2.DOCX]

**Supplementary Table 2.** Sensitivity analyses of NEDA-3 excluding Week 12 MRI activity. NEDA-3 was defined as no confirmed relapses, no Gd+ T1 lesions, no new or enlarging T2 lesions, and no 12-week CDP. Pooled post hoc sensitivity analyses excluded the Week 12 MRI data, which evaluated Gd+ T1 lesions only. Modified intention-to-treat population. CDP, confirmed disease progression; EDSS, Expanded Disability Status Scale; Gd+, gadolinium-enhancing; MRI, magnetic resonance imaging; NEDA-3, 3-parameter no evidence of disease activity. ^a^Logistic regression model with baseline adjustments, treatment, study (for pooled analysis), region, baseline EDSS score strata, plus log-transformed baseline MRI lesion counts (T1 nonenhancing, T2, Gd+ lesions). ^b^Participants may have > 1 component of evidence of disease activity. ^c^Independent Relapse Adjudication Committee confirmed.

| **Weeks 0–48** | Ublituximab  (n = 527) | Teriflunomide (n = 528) | Odds ratio^a^ (95% CI) | *p* value |
| --- | --- | --- | --- | --- |
| Participants with NEDA-3,^b^ n (%) | 262 (49.7) | 112 (21.2) | 4.79 (3.58, 6.41) | < 0.0001 |
| Participants free of relapse,^c^ n (%) | 480 (91.1) | 437 (82.8) |  | |
| Participants free of 12-week CDP | 514 (97.5) | 514 (97.3) |  |  |
| Participants free of Gd+ T1 lesions, n (%) | 518 (98.3) | 311 (58.9) |  |  |
| Participants free of new or enlarging T2 lesions, n (%) | 295 (56.0) | 135 (25.6) |  |  |
|  | | |  |  |
| **Weeks 0–96** | Ublituximab  (n = 520) | Teriflunomide  (n = 524) | Odds ratio^a^ (95% CI) | *p* value |
| Participants with NEDA-3,^b^ n (%) | 234 (45.0) | 67 (12.8) | 7.09 (5.12, 9.83) | < 0.0001 |
| Participants free of relapse,^c^ n (%) | 450 (86.5) | 384 (73.3) |  | |
| Participants free of 12-week CDP | 492 (94.6) | 492 (93.9) |  |  |
| Participants free of Gd+ T1 lesions, n (%) | 511 (98.3) | 271 (51.7) |  |  |
| Participants free of new or enlarging T2 lesions, n (%) | 287 (55.2) | 99 (18.9) |  |  |
